# Evidence for Negative Effects of Elevated Intra-Abdominal Pressure on Pulmonary Mechanics and Oxidative Stress

**DOI:** 10.1155/2015/612642

**Published:** 2015-01-20

**Authors:** I. Davarcı, M. Karcıoğlu, K. Tuzcu, K. İnanoğlu, T. D. Yetim, S. Motor, K. T. Ulutaş, R. Yüksel

**Affiliations:** ^1^Department of Anesthesiology, Faculty of Medicine, Mustafa Kemal University, Hatay, Turkey; ^2^Department of Thoracic Surgery, Faculty of Medicine, Mustafa Kemal University, Hatay, Turkey; ^3^Department of Biochemistry, Faculty of Medicine, Mustafa Kemal University, Hatay, Turkey

## Abstract

*Objective*. To compare the effects of pneumoperitoneum on lung mechanics, end-tidal CO_2_ (ETCO_2_), arterial blood gases (ABG), and oxidative stress markers in blood and bronchoalveolar lavage fluid (BALF) during laparoscopic cholecystectomy (LC) by using lung-protective ventilation strategy. *Materials and Methods*. Forty-six patients undergoing LC and abdominal wall hernia (AWH) surgery were assigned into 2 groups. Measurements and blood samples were obtained before, during pneumoperitoneum, and at the end of surgery. BALF samples were obtained after anesthesia induction and at the end of surgery. *Results*. Peak inspiratory pressure, ETCO_2_, and pCO_2_ values at the 30th minute were significantly increased, while there was a significant decrease in dynamic lung compliance, pH, and pO_2_ values in LC group. In BALF samples, total oxidant status (TOS), arylesterase, paraoxonase, and malondialdehyde levels were significantly increased; the glutathione peroxidase levels were significantly decreased in LC group. The serum levels of TOS and paraoxonase were significantly higher at the end of surgery in LC group. In addition, arylesterase level in the 30th minute was increased compared to baseline. Serum paraoxonase level at the end of surgery was significantly increased when compared to AWH group. *Conclusions*. Our study showed negative effects of pneumoperitoneum in both lung and systemic levels despite lung-protective ventilation strategy.

## 1. Introduction

The increasing number of indications of laparoscopic surgery, which is the gold standard approach in several diagnostic and therapeutic procedures, means that anesthesiologists need to have a better understanding of the physiological effects and potential complications of pneumoperitoneum [1]. Laparoscopic surgery, a minimal invasive technique, has substantial effects on the hemodynamic and respiratory system, even in healthy individuals, although it has many advantages compared to conventional open surgical techniques. These pathophysiological effects result in an increased risk of perioperative and postoperative complications in elderly patients with impaired cardiac and pulmonary functions [2, 3].

During laparoscopy, increasing intra-abdominal pressure (IAP) with abdominal CO_2_ insufflation causes ischemia through splanchnic vasoconstriction and subsequent reperfusion injury through deflation [2]. Moreover, hypercarbia and acidosis can occur because of ventilation-perfusion mismatch caused by impaired gas exchange due to increased IAP or absorption of insufflated CO_2_. Hypercarbia and acidosis affect serum oxidative stress markers and lead to altered hemodynamics [4, 5].

In this prospective study, we evaluated the effects of pneumoperitoneum at an IAP level (<12 mmHg) accepted to be clinically safe on lung mechanics, end-tidal CO_2_ (ETCO_2_), arterial blood gases, and oxidative stress markers in blood and bronchoalveolar lavage fluid (BALF) during laparoscopic cholecystectomy (LC) using a lung-protective ventilation strategy.

## 2. Methods

The study was approved by the Local Human Research Board of Mustafa Kemal University, Medicine School. All the patients gave written informed consent before participation in the study. Forty-six patients aged 18–65 years with ASA physical statuses I-II who were scheduled for laparoscopic cholecystectomy and abdominal wall hernia (AWH) surgery were assigned to 2 groups, with 23 patients in each group. Patients with cardiac, pulmonary, renal, hepatic, metabolic, endocrine, and inflammatory diseases, such as autoimmune and infectious diseases, as well as a history of malignant disease, were excluded. Those with acute cholecystitis or those who were morbidly obese (body mass index [BMI] > 25 kg/m^2^), had a history of smoking or alcohol consumption, and had used any drug with antioxidant properties within the previous 48 hours or lipid-lowering agents were also excluded. Demographic characteristics, including age, gender, weight, and height, as well as clinical data and the duration of anesthesia and operation, were recorded in all the patients. All the patients underwent routine clinical monitoring throughout the surgery, including electrocardiography and heart rate monitoring and noninvasive measurements of blood pressure, peripheral oxygen saturation (SpO_2_), and ETCO_2_ (Datex-Ohmeda and ADU, S/5, Helsinki, Finland). The patients were premedicated before the induction of anesthesia with midazolam 2 mg/kg. General anesthesia was administered using lidocaine 40 mg (Biologici Italia Laboratories, Milan, Italy), propofol 2 mg/kg (Propofol; Fresenius Kabi, Austria), and fentanyl 1–1.5 *μ*g/kg (Abbott Laboratoires, Abbott Park, Illinois, USA). Endotracheal intubation was achieved after muscle relaxation by rocuronium 0.6 mg/kg (Rocuroniumbromür; Organon, Oss, Holland).

Anesthesia was maintained by 2-3% (1–1.5 MAC) sevoflurane in a 50%/50% (v/v) oxygen-air mixture. A fentanyl bolus (50 *μ*g) was used if additional analgesia was required during the surgery. In addition, to maintain complete neuromuscular block, an additional rocuronium bolus (0.15 mg/kg) was given at 30 min intervals or when needed.

The patients' lungs were mechanically ventilated with a volume-controlled ventilation mode using a lung-protective ventilation strategy (40% oxygen in air at a frequency of 10–12 breaths per min), and the tidal volume was adjusted to maintain an ETCO_2_ level of 35–40 mmHg (VTffi: 8 mL/kg^−1^; I : E ratio 1 : 2; PEEP: 4 cmH_2_O; peak airway pressure: 15–20 cmH_2_O) [6].

CO_2_ pneumoperitoneum was performed by automatic insufflators (Karl Storz, Tuttlingen, Germany), and IAP of 10–12 mmHg was held at low speed (2 L/min) during the laparoscopic surgery.

The baseline peak inspiratory pressure (PIP), plateau pressure (PP), and ETCO_2_ were measured, and the dynamic lung compliance (C_dyn_) was calculated using the TV/PIP 10 min after the induction of anesthesia. All the measurements were then repeated 30 min after pneumoperitoneum in the LC group, 30 min after the skin incision in the AWH group, and at the end of the surgery before skin closure in both groups (after pneumoperitoneum in the LC group).

To analyze the total antioxidant status (TAS), total oxidant status (TOS), paraoxonase (PON1), arylesterase, glutathione peroxidase (GSH-Px), and malondialdehyde (MDA) levels, blood samples were drawn before the induction of anesthesia (baseline), 30 min after pneumoperitoneum in the LC group, 30 min after the incision in the AWH group (second blood samples), and at the end of the surgery before skin closure in both groups (third blood samples). Arterial blood gases were also analyzed at the same time as the blood samples.

Alveolar cells were obtained after anesthesia induction as a baseline and at the end of the surgery before extubation via the nonbronchoscopic blind bronchoalveolar lavage catheter technique described by Koksal et al. [7]. Heparinized blood and alveolar lavage samples were centrifuged at 1500 rpm for 10 min within 45 min after collection. The supernatant fluid was then transferred to Eppendorf tubes and immediately stored at −80°C until testing.

### 2.1. Measurement of MDA

MDA was measured in serum and BALF samples by the method described by Ohkawa et al. where the substrate reacts with thiobarbituric acid, and the results were expressed as nmol/L [8].

### 2.2. Measurement of GSH-Px

GSH-Px was measured in serum and BALF samples. Enzyme activities in BALF were expressed as U/L. GSH-Px activity was measured by the method of Paglia and Valentine [9]. Enzyme activities were expressed as U/L.

### 2.3. Measurement of Total Antioxidant Status

Serum TAS was determined using the novel automated measurement method by Erel, with commercially available kits (Rel Assay Diagnostic, Turkey). Antioxidants in the sample turned from dark blue-green colored ABTS radical to colorless reduced ABTS form. The change of absorbance at 660 nm denotes the total antioxidant level of the sample. The assay was calibrated with a stable antioxidant standard solution, Trolox Equivalent, which is a vitamin E analogue [10]. The results were expressed as mmol Trolox equivalent/L.

### 2.4. Measurement of Total Oxidant Status

Serum TOS was determined using the novel automated measurement method by Erel [11]. Oxidants present in the sample oxidize the ferrous ion-chelator complex to ferric ion. The oxidation reaction is prolonged by enhancer molecules, which are abundantly present in the reaction medium. The ferric ion makes a colored complex with chromogen in an acidic medium. The color intensity, which can be measured spectrophotometrically, is related to the total amount of oxidant molecules present in the sample. The assay is calibrated with hydrogen peroxide and the results are expressed in terms of the micromolar hydrogen peroxide equivalent per liter (*μ*mol H_2_O_2_ Equiv./L).

### 2.5. Measurements of Paraoxonase and Arylesterase Activities

Paraoxonase and arylesterase activities were measured with commercially available kits (Rel Assay Diagnostic, Turkey). Fully automated paraoxonase activity measurement was performed either in the presence (salt-stimulated) or in the absence of NaCl. The paraoxon hydrolysis rate (diethyl-p-nitrophenyl phosphate) was measured by monitoring increased absorption at 412 nm at 37.8°C. The amount of generated p-nitrophenol was calculated from the molar absorption coefficient at pH 8.5, which was 18.290/M per cm. Paraoxonase activity was expressed as U/L serum. The coefficient of variation for individual samples was 1.8%. Serum arylesterase activity was determined by the presence of phenol following the reactions of phenylacetate. The molar extinction coefficient of phenol was 4,000 M^−1^ cm^−1^; the results were expressed as kU/L [12].

### 2.6. Statistical Analysis

All data were analyzed using SPSS (Statistical Package for the Social Science, version 14.0, SSPS Inc., Chicago, IL, USA) software. The Kolmogorov-Smirnov and Shapiro-Wilk tests were used as tests of normality, and Levene's homogeneity of variance test was applied to all the variables. Significant statistical differences in the mean values of the variables between the groups were examined using Student's *t*-test or the Mann-Whitney *U* test. A paired sample *t*-test or Wilcoxon's test was utilized for dependent variables. *P* values less than 0.05 were considered statistically significant.

## 3. Results

There was no significant difference between the groups regarding demographic data, ASA classification, and duration of surgery and anesthesia (Table 1). Throughout the surgery, all the patients were hemodynamically stable in terms of heart rate, blood pressure, and SpO_2_. Thus, none of the patients were excluded (data not shown).

In LC group, there was a significant increase in the PIP, ETCO_2_, and pCO_2_ values (*P* < 0.023, *P* < 0.0001, and *P* < 0.001, resp.), and there was a significant decrease in the C_dyn_, pH, and pO_2_ values at 30 min compared to baseline values (*P* < 0.0001, *P* < 0.001, and *P* < 0.0001, resp.) (Table 2).

The PIP, C_dyn_, and ETCO_2_ returned to baseline values at the end of the surgery, and these showed a significant difference compared to the values obtained during pneumoperitoneum (*P* < 0.0001, *P* < 0.0001, and *P* < 0.0001, resp.). Changes observed in arterial blood gases persisted during this period. The pCO_2_ values were increased and the pH values were decreased, and the differences were significant when compared to the baseline values (*P* < 0.002 and *P* < 0.003, resp.). The level of pO_2_ was decreased, and this decrease was significant when compared to both the baseline and pneumoperitoneum periods (*P* < 0.0001 and *P* < 0.0001, resp.) (Table 2).

In the AWH group, no significant difference was observed in the PIP, C_dyn_, ETCO_2_, pH, pCO_2_, and pO_2_ values (*P* > 0.05). In addition, no significant difference was observed in the PP values in both groups (*P* > 0.05) (Table 2).

In the LC group, the PIP and ETCO_2_ values were significantly increased (*P* < 0.048 and *P* < 0.0001, resp.), whereas the C_dyn_ values were significantly decreased when compared to the AWH group (*P* < 0.001). In the LC group, there was a significant decrease in the pH (*P* < 0.001 and *P* < 0.039, resp.) and pO_2_ values (*P* < 0.0001 and *P* < 0.041, resp.) and a significant increase in pCO_2_ at 30 min and at the end of surgery when compared to the AWH group (*P* < 0.002 and *P* < 0.020, resp., Table 2).

**Figure 1:**
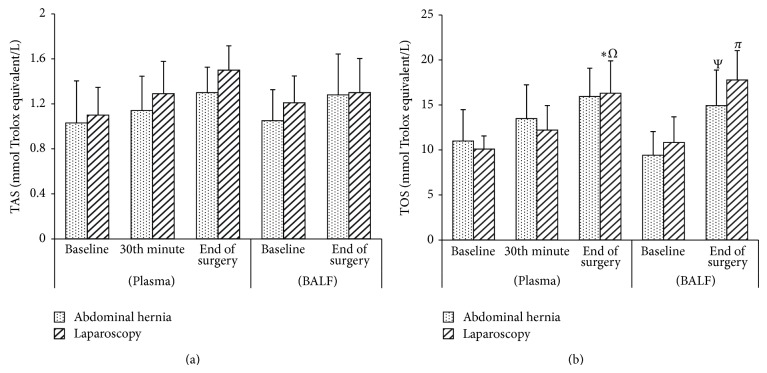
Pre- and perioperative TAS (total antioxidant status) (a) and TOS (total oxidant status) (b) levels of the groups in plasma and BALF. Data are expressed as mean ± standard error. ^*^ *P* < 0.001 compared to plasma baseline, ^Ω^ *P* < 0.05 compared to 30th minute, ^Ψ^ *P* < 0.05 compared to BALF baseline, ^*π*^ *P* < 0.05 compared to BALF baseline.

**Figure 2:**
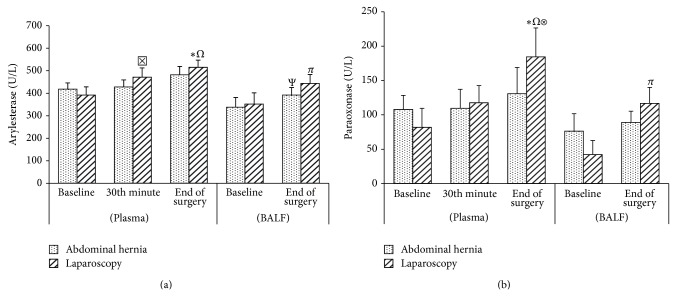
Pre- and perioperative ARES (arylesterase) (a) and PON (paraoxonase) levels (b) levels of the groups in plasma and BALF. Data are expressed as mean ± standard error. ^*^ *P* < 0.001 compared to plasma baseline, ^Ω^ *P* < 0.05 compared to 30th minute, ^Ψ^ *P* < 0.05 compared to BALF baseline, ^*π*^ *P* < 0.01 compared to BALF baseline, ^⊠^ *P* < 0.001 compared to plasma baseline, ^⊗^ *P* < 0.01 compared to laparoscopy versus abdominal wall hernia.

**Figure 3:**
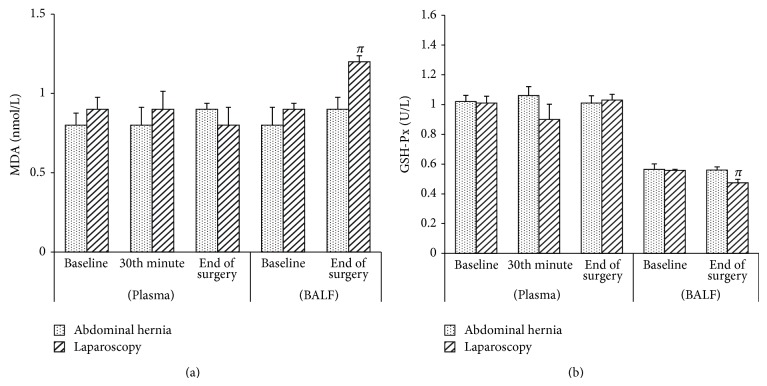
Pre- and perioperative MDA (malondialdehyde) (a) and GSH-Px (glutathione peroxidase) (b) levels of the groups in plasma and BALF. Data are expressed as mean ± standart error. ^*π*^ *P* < 0.05 compared to BALF baseline.

In both groups, no significant difference was observed in the TAS levels between BALF samples obtained at the end of surgery and those obtained after induction (Figure 1(a)). In the LC group, a significant increase was observed in TOS, arylesterase, paraoxonase, and MDA levels (*P* < 0.003, *P* < 0.010, *P* < 0.001, and *P* < 0.021, resp.) at the end of surgery when compared to baseline values and those obtained after induction, whereas there was a significant decrease in GSH-Px levels (*P* < 0.020) in the BALF samples (Figures 1(b), 2(a), 2(b), 3(a), and 3(b)). In the AWH group, similar changes were observed in TOS and arylesterase levels (*P* < 0.025 and *P* < 0.003, resp.), whereas no significant difference was observed in MDA, GSH-Px, and paraoxonase levels (Figures 1(b), 2(a), 2(b), 3(a), and 3(b)).

No significant difference was observed between the groups in TAS, TOS, paraoxonase, arylesterase, MDA, and GSH-Px levels in the BALF (Figures 1, 2, and 3).

There was no significant difference in the serum levels of TAS, MDA, and GSH-Px in the LC group (Figures 1(a), 3(a), and 3(b)). There was a significant increase in the TOS (*P* < 0.001 and *P* < 0.05, resp.) and paraoxonase (*P* < 0.001 and *P* < 0.04, resp.) in serum at the end of surgery compared to baseline and at 30 min (Figures 1(b) and 2(b)). There was also a significant increase in arylesterase levels at the end of surgery compared to baseline and at 30 min, in addition to 30 min compared to baseline values (*P* < 0.001, *P* < 0.028, and *P* < 0.001, resp.) (Figure 2(a)).

In the AWH group, no significant difference was observed in serum TAS, TOS, arylesterase, paraoxonase, MDA, and GSH-Px levels (Figures 1, 2, and 3). When the groups were compared, only serum paraoxonase levels at the end of surgery after deflation were increased in the LC group when compared to those of the AWH group (*P* < 0.014) (Figure 2(b)).

## 4. Discussion

The results of our study showed that CO_2_ pneumoperitoneum markedly increased PIP, pCO_2_, and ETCO_2_ levels and decreased C_dyn_, pO_2,_ and pH levels when compared to those of the AWH group. In the LC group, the increase in pCO_2_ and the decrease in pO_2_ and pH persisted, although all respiratory parameters returned to baseline values after deflation. The biochemical results for BALF and the serum samples supported the abovementioned effects.

Local-systemic inflammatory, endocrine-metabolic, and immunological responses observed in surgical procedures are associated with the volatile anesthetics used, mechanical ventilation, and surgical stress [13]. Patients undergoing laparoscopic cholecystectomy due to cholelithiasis are exposed to oxidative stress caused by pneumoperitoneum and the chronic pattern of the disease, in addition to the abovementioned effects of general anesthesia [14]. CO_2_ pneumoperitoneum leads to several pathophysiological alterations in many systems. During laparoscopy, the most extensive changes are observed in the cardiovascular system. The changes observed during CO_2_ pneumoperitoneum are mainly caused by hypercarbia, acidosis, and elevated IAP [5]. Acidosis caused by the direct effect of the CO_2_ gas used plays a significant role in the intraoperative pulmonary inflammatory response to pneumoperitoneum. Hypercarbia and acidosis occur due to the passage of CO_2_ with high solubility and peritoneal absorption [15]. In addition, impaired intrapulmonary distribution of CO_2_ and decreased elimination of the gas occur due to increased intrathoracic pressure [4, 5, 16]. In general, respiratory acidosis is well tolerated by healthy individuals, and the blood pH is normalized by the buffering systems of body. This recycling can be challenging or occasionally impossible in patients with comorbid cardiorespiratory disease and in surgical procedures with prolonged periods of elevated IAP. Lower IAPs (10 mmHg) and shorter durations of pneumoperitoneum, as well as hyperventilation and sufficient PEEP support, can be beneficial in such patients. Open surgery should be considered if the abovementioned measures fail [5, 17].

Elevated IAP can also change respiratory mechanics. One study reported a decrease of 27% in respiratory system compliance and an increase of 35% in peak inspiratory pressure in cases where pneumoperitoneum was achieved with a pressure level of 15 mmHg [18]. These alterations return to normal control levels within 90 min after cessation of pneumoperitoneum. Laparoscopic cholecystectomy in the head-up tilt position is considered more appropriate for respiration. Moreover, alterations in compliance might be regulated swiftly after deflation in the head-up position rather than the head-down position [3]. Prolonged pneumoperitoneum can cause alterations in pulmonary compliance, which can take longer time to return to normal.

Ortiz-Oshiro et al. suggested that restriction of pressure by 12 mmHg and short surgery time are the most important factors to prevent potential oxidative injury in laparoscopic surgery [19].

In our study, IAP lower than 12 mmHg, which is clinically accepted, was used to provide adequate ventilation and oxygenation and to minimize respiratory changes. In addition, to prevent hypercapnia and acidosis during the surgery, the respiratory rate was adjusted, with the ETCO_2_ being lower than 35 mmHg. By these measures, increased PIP and ETCO_2_ levels and decreased C_dyn_ during pneumoperitoneum returned to baseline levels after cessation of pneumoperitoneum. However, the impairment in blood gases persisted.

Kazama et al. showed that CO_2_ production increased by 50% and CO_2_ elimination continued even after desufflation [20]. In another study, CO_2_ levels returned to normal after deflation increased again 30 min after extubation [21].

The effects of elevated IAP due to pneumoperitoneum on the lung are not limited to the abovementioned factors. Normalization of blood flow by desufflation causes reperfusion in ischemic organs following ischemia due to a decrease in the blood flow of intra-abdominal organs by 10–80% during laparoscopy [22, 23]. Free oxygen radicals resulting from ischemia-reperfusion (I/R) injury due to laparoscopic procedures cause oxidative injury in distant organs, particularly lungs, other than splanchnic organs [24, 25]. There are a limited number of studies of the effects of pneumoperitoneum on the lungs, although numerous studies have investigated oxidative damage in intra-abdominal organs caused by pneumoperitoneum [7, 25, 26]. In these studies, there is no consensus on a valid marker of peroxidation or a method of determining oxidative stress, despite the use of several parameters of oxidative stress [2, 27].

In our study, increased TOS levels in BALF were detected in both groups as a result of mechanical ventilation and the volatile anesthetics used in general anesthesia, as well as surgical stress. High serum TOS levels indicating splanchnic ischemia caused by the increased IAP due to pneumoperitoneum persisted in systemic circulation during the reperfusion period after deflation. Moreover, in patients in the LC group, increased MDA levels in BALF and increased GSH-Px consumption were observed at the end of the surgery in patients in the LC group.

Free oxygen radicals, which are continuously produced as a result of aerobic metabolism, absorb H^+^ ions along lipid membranes. Lipid peroxidation products, which are produced by a number of reactions, lead to the formation of highly reactive aldehydes and damage membrane lipids. Measurement of MDA levels is the most important marker of free radical-mediated lipid peroxidation [28, 29]. Increased MDA levels due to ischemic damage in laparoscopic surgery have been demonstrated in many experimental and clinical trials [7, 30]. Glantzounis et al. reported that there was an increase in lipid peroxidation products and a decrease in endogenous antioxidants in the early postoperative period in laparoscopic surgery when compared to open cholecystectomy [31]. GSH-Px, one of the most important sources of antioxidant defense, plays a role in the removal of free oxygen radicals to protect the structure and function of biological membranes. In a rat model, Pross et al. found decreased GSH-Px levels accompanying marked lipid peroxidation in lungs during laparoscopy with CO_2_ pneumoperitoneum when compared to laparoscopy without gas insufflation [26]. These authors reported that the oxidative stress contributed to impairment of pulmonary functions in laparoscopic procedures with CO_2_ insufflation. In rats in which experimental pancreatitis was induced, Polat et al. detected increased MDA and decreased glutathione levels in rats that underwent laparoscopy with various pressure levels when compared to an open laparotomy group [32]. The authors suggested that laparoscopy increased the severity of pancreatitis. Our results support the presence of oxidative stress in the lung in the LC group.

Previous studies reported several biomarkers of decreased antioxidant enzyme levels, as well as compensatory and regulatory activity of antioxidants in oxidative stress [27, 33]. Some showed that the activity and the expression of paraoxonase with well-known antioxidant properties were decreased in oxidative stress [27, 34]. Paraoxonase, an ester hydrolase, exhibits dyazoxonase and Ca+2 dependent serum esterase activities, in addition to arylesterase and paraoxonase activities [35, 36]. It has been reported that paraoxonase activity is decreased in chronic inflammation, several diseases, and some cancer types [27, 37–39]. PON1 is helpful in monitoring the antioxidant defence system as well as TAS, TOS, MDA, and GSH-Px, in I/R injury [40, 41]. Yalcın et al. showed increased oxidant levels and decreased arylesterase activities in cholelithiasis, which is considered a chronic inflammatory process [14]. In our study, baseline serum paraoxonase and arylesterase levels were lower in the LC group compared to the AWH group. Therefore, the presence of cholelithiasis appears to have contributed to oxidative stress in the LC group by inducing inflammation through the production of reactive oxygen species.

In the perioperative period, the gradual increase in serum paraoxonase levels in the LC group compared to those in the AWH group has been attributed to a protective response to the early phase of oxidative damage caused by I/R injury. The increase is also thought to be associated with stimulation of the antioxidant defense system by increased oxidative stress. Uzar et al. reported that cerebral paraoxonase levels were increased in cerebral I/R injury [40]. Topsakal et al. reported increased paraoxonase levels in spinal cord injury [41]. Our results are in agreement with the literature.

The present study demonstrated clear effects of pneumoperitoneum in both serum and BALF oxidative parameters simultaneously with respiratory changes. In our study, acidosis and oxidative stress observed during pneumoperitoneum persisted following deflation at the end of the surgery in the laparoscopy group, although an IAP lower than 12 mmHg and a lung-protective ventilation strategy were used to prevent hypercapnia and acidosis. Undesirable effects of pneumoperitoneum on pulmonary functions can be tolerated in healthy individuals without development of hypoxemia or hypercapnia, whereas it can cause postoperative pulmonary complications in patients with comorbid pulmonary disease [5, 28].

Given the presence of chronic oxidative stress in patients with cholelithiasis, anesthesiologist should be aware of changes in respiratory dynamic and oxidative stress caused by pneumoperitoneum. In addition to the abovementioned measures, cardiovascular and pulmonary functions should be carefully monitored to prevent postoperative pulmonary complications, particularly in those with comorbid disease. Further studies are needed to develop novel anesthetic approaches and surgical techniques to control and limit pneumoperitoneum-related changes in pulmonary functions and oxidative stress.

## Figures and Tables

**Table 1:** Clinical characteristics of the patients and operation-anesthesia durations of groups.

	Laparoscopy group	Abdominal wall hernia group	*P*
Age (year)	42.61 ± 3.08	43.30 ± 2.61	0.414
Weight (kg)	66.91 ± 5.61	67.48 ± 5.88	0.741
Sex (female/male)	14/9	12/11	0.562
ASA I/II	13/10	11/12	0.565
Operation duration (min)	58.09 ± 2.57	58.61 ± 3.15	0.542
Anesthesia duration (min)	67.70 ± 2.81	68.78 ± 2.96	0.210

Data are expressed as mean ± standard deviation.

**Table 2:** Pre- and perioperative levels of respiratory parameters and measurements of AKG in patients who underwent laparoscopic cholecystectomy and abdominal wall hernia.

	Laparoscopy group			Abdominal wall hernia group		
	Baseline	30th minute	End of surgery	Baseline	30th minute	End of surgery
PIP	20 (16–32)	22 (19–33)^a,∗^	20 (17–32)^c^	23 (20–27)	23 (18–28)	23 (20–28)
PLT	20 (11–33)	21 (10–36)	20 (12–33)	19 (15–27)	20 (14–27)	20 (15–25)
C_dyn_	28.50 (22.72–38.55)	21.53 (17.30–29.41)^a,∗^	28.50 (22.70–38.55)^c^	26.31 (20.50–35.61)	26.30 (20.53–35.62)	26.31 (20.52–35.60)
pO_2_	202 (122–268)	125 (53–226)^a,∗^	162 (110–250)^b,c,∗^	185 (102–279)	189 (120–270)	185 (123–340)
pCO_2_	38 (31–52)	45 (34–59)^a,∗^	44 (27–60)^b,∗^	41 (32–52)	39 (32–53)	40 (19–52)
pH	7.39 (7.31–7.49)	7.34 (7.24–7.50)^a,∗^	7.35 (7.26–7.48)^b,∗^	7.39 (7.33–7.44)	7.40 (7.29–7.46)	7.38 (7.30–7.55)
ETCO_2_	31 (30–33)	35 (34–36)^a,∗^	31 (30–32)^c^	31 (29–33)	32 (28–34)	31 (29–33)

Data are expressed as median (min–max).

Statistically significant differences (*P* < 0.05) were noted as follows: a; baseline versus 30th minute, b: baseline versus end of surgery, c; 30th versus end of surgery.

∗; Laparoscopy group versus abdominal wall hernia group.

PIP: peak in respiratory pressure, PLT: plateau pressure, C_dyn_: dynamic lung compliance, ETCO_2_: end-tidal CO_2_.
